# Study on Micro-Displacement Mechanism and Reservoir Compatibility of Soft Dispersed Microgel

**DOI:** 10.3390/gels9030177

**Published:** 2023-02-23

**Authors:** Yinzhu Ye, Yang Liu, Baoshan Guan, Zhe Yang, Lipeng He, Peiwen Xiao, Xiaocong Wang, Shichao Li

**Affiliations:** 1 Research Institute of Petroleum Exploration and Development (RIPED), China National Petroleum Corporation, Beijing 100083, China; 2 Key Laboratory of Nano Chemistry (KLNC), China National Petroleum Corporation, Beijing 100083, China; 3 Exploration and Development Research Institute of Southwest Oil and Gas Field Company, China National Petroleum Corporation, Chengdu 610041, China; 4 No. 1 Oil Production Plant of North China Oil and Gas Company, SINOPEC, Xianyang 712034, China

**Keywords:** soft dispersed microgel, visualization experiment, displacement mechanism, reservoir compatibility, EOR

## Abstract

Polymer flooding is a key technology for improving reservoir heterogeneity around the world, and it has made great progress. However, the traditional polymer has many shortcomings in the theory and application, which causes the efficiency of polymer flooding to gradually decrease and secondary reservoir damage after a long period of polymer flooding. In this work, a novel polymer particle (soft dispersed microgel, SMG) is used as the research object to further investigate the displacement mechanism and reservoir compatibility of SMG. The visualization experiments of the micro-model prove that SMG has excellent flexibility and can be highly deformable to realize deep migration through the pore throat smaller than SMG itself. The visualization displacement experiments of the plane model further show that SMG has a plugging effect, which makes the displacing fluid flow into the middle and low permeability layers, improving the recovery of these layers. The compatibility tests show that the optimal permeability of the reservoir for SMG-μm is 250–2000 mD, and the corresponding matching coefficient range is 0.65–1.40. For SMG-mm^−^, its corresponding optimal permeabilities of reservoir and matching coefficient are 500–2500 mD and 1.17–2.07, respectively. The comprehensive analysis demonstrates that the SMG has excellent ability of the water-flooding swept control and compatibility with reservoirs, having the potential to solve the problem of traditional polymer flooding.

## 1. Introduction

The types of oilfields in China are complex and diverse, most of which are continental sedimentary and characterized by severe heterogeneity [1,2,3,4]. The number of oil reservoirs developed by water flooding accounts for more than 92% of the total reservoirs in China. The majority of oilfields of China National Petroleum Corporation (CNPC) have entered the period of high water-cut and extra high water-cut except for Changqing. The remaining recoverable reserves of these high water-cut and extra high water fields account for more than 60%, and their production accounts for more than 70% of the total production. Therefore, improving the effect of water-flooding development is of great significance to the strategic goal and national security of stable production of 100 million tons of CNPC.

Polymer flooding is the main method for ineffective or inefficient water flooding in high water-cut and extra high water-cut oilfields [5,6,7,8,9]. Polymer is developed from inorganic profile control agents such as clay, fly ash, and cement to organic gel profile control agents in the 1970s. Although polymer flooding has made great progress [10,11,12,13,14,15,16,17,18], due to its small injection volume and small treatment radius, it can only play a certain role in the longitudinal heterogeneity near-well zone, and it is only a production measure, strictly speaking. In order to solve the limitation that polymer flooding can only be used in the near-well area. Many scholars try to conduct “high-dose profile control”, also known as “deep profile control” [19,20,21,22,23], but the treatment radius is often no more than 10–20 m and the treatment cost is high. In addition, with the increase of water-cut in oilfields, the water-flooding contradiction changes from the shallow reservoir to the deep reservoir, or even the entire reservoir flow field. The traditional polymer flooding, including profile control and water shut-off technology, can only achieve the purpose of increasing the production of a single well and cannot fundamentally solve the problem of improving recovery in a high water-cut period.

In order to solve these problems, SMG-based water-flooding swept control technology is established by Professor Wu Xingcai, Research Institute of Petroleum Exploration, and Development of CNPC [24]. This technique can effectively sweep the whole flow field through a process of temporary plugging-passing-turning-plugging-passing of a large number of SMG in the whole reservoir [25,26,27]. This technology has been used in many reservoirs with different temperatures (28–126 °C), different viscosities (4–165 mPa·s), different salinities (2000–20 × 10^4^ mg/L), different water-cuts (80–98%), and different production degrees (14–48%) in North China, Xinjiang, Liaohe, Qinghai, Dagang, and so on, and it has achieved obvious results [28,29,30,31,32,33]. However, the oil displacement mechanism of SMG is not clear enough, and the exact matching between SMG and reservoirs is lacking.

In this work, the oil displacement mechanism of SMG is further studied, and the effectiveness of SMG-based water-flooding swept control technology is confirmed by the visualization experiments. Besides, the matching relationship between SMG and reservoirs has been determined by the displacement experiments of cores. The comprehensive analysis demonstrates that the SMG has excellent ability of water-flooding swept control and compatibility with reservoirs, having the potential to solve the problem of the traditional polymer flooding.

## 2. Results and Discussions

### 2.1. Swelling Property of SMG

The swelling multiple is a representation of the SMG with hydration swelling property. The initial median particle size of SMG is *d*_0_, which reflects the median particle size before hydration swelling. After 7 days of SMG hydration swelling, the measured median particle size of SMG is *d_max_*. The test results show that the expansion multiple (*B*) of SMG particles is about 3–8 times, and the expansion multiple of SMG-μm is larger than that of SMG-mm^−^, as shown in Table 1. The concentration of SMG aqueous dispersion is 3000 mg/L. The experimental temperature is 60 °C.

In general, the polymer solution, the melt fluid of the discrete system of colloidal particles, are all non-Newtonian fluids. The swelling rate of SMG hydration belongs to the kinetic problem of water absorption of polymer materials. Superabsorbent gels belong to elastic gels, and they follow the general law of elastic gels expansion kinetics.

(1) $$\frac{\delta u}{\delta t}=D\frac{\delta^{2}u}{\delta^{2}r}$$

(2) $$D=k/f_{r}$$

where δuδt is the diffusion velocity of the gel unit. δ2uδ2r is the second derivative of the change in the radius of the diffusion element. The diffusion coefficient *D* is the elasticity of the mesh. *K* is the ratio of the mesh to the viscosity coefficient *f_r_* of the solvent.

Equation (1) is derived by Japanese researchers based on Flory’s theory according to Fickian diffusion’s second law.

Equation (2) is the diffusion equation. By solving this equation, Equation (3) can be obtained, which can explain the relationship between characteristic swelling time *t* and characteristic linear size *L*.

(3) $$t\propto \frac{L^{2}}{D}$$

This is the kinetic equation for the volume expansion of the gel in the solvent. From this equation, it can be seen that the smaller the particle size of the gel, the faster the swelling. It should be noted that due to the different shapes and ionization states of gels, they should be adjusted in practical applications. This mechanism also further explains why the expansion times of hydration measured by SMG-μm are greater than that measured by SMG-mm^−^.

**Figure 1:**
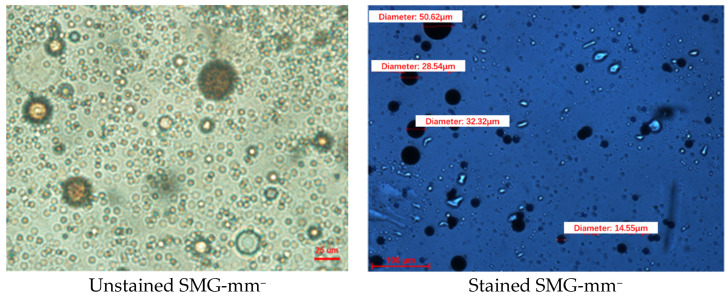
Micromorphology of SMG-mm^−^ under microscope.

The observation results of SMG morphology show that SMG particles are spherical with good sphericity, as shown in Figure 1. Then, for the same sample, the size of SMG particles after purification dyeing are not different from those without dyeing, as shown in Figure 1.

### 2.2. Rheological Property of SMG Aqueous Dispersion

**Figure 2:**
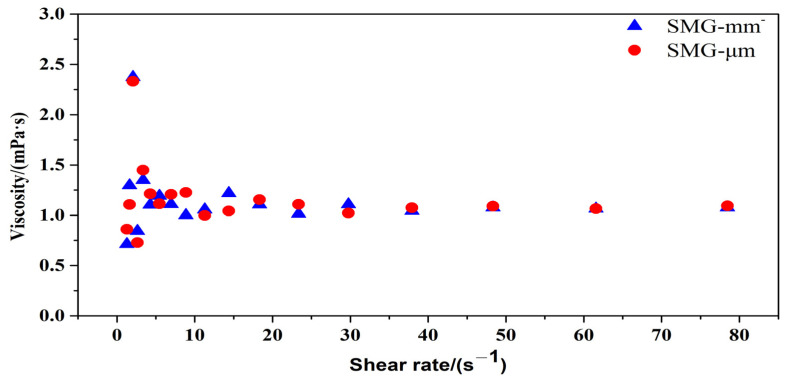
The relationship between the apparent viscosity of SMG aqueous dispersion and shear rate.

The rheological property of SMG aqueous dispersion with a concentration of 3000 mg/L is tested. The relationship between the apparent viscosity of SMG aqueous dispersion and shear rate is shown in Figure 2. The apparent viscosity fluctuates when the shear rate is 1–10 s^−1^. When the shear rate is 10–80 s^−1^, the apparent viscosity is unstable and slightly decreased, showing a weak pseudoplastic character.

**Figure 3:**
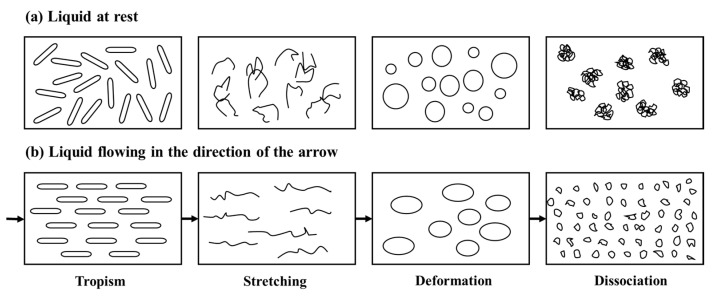
Schematic diagram of the flexible particle system stationary and flowing through the pore.

**Figure 4:**
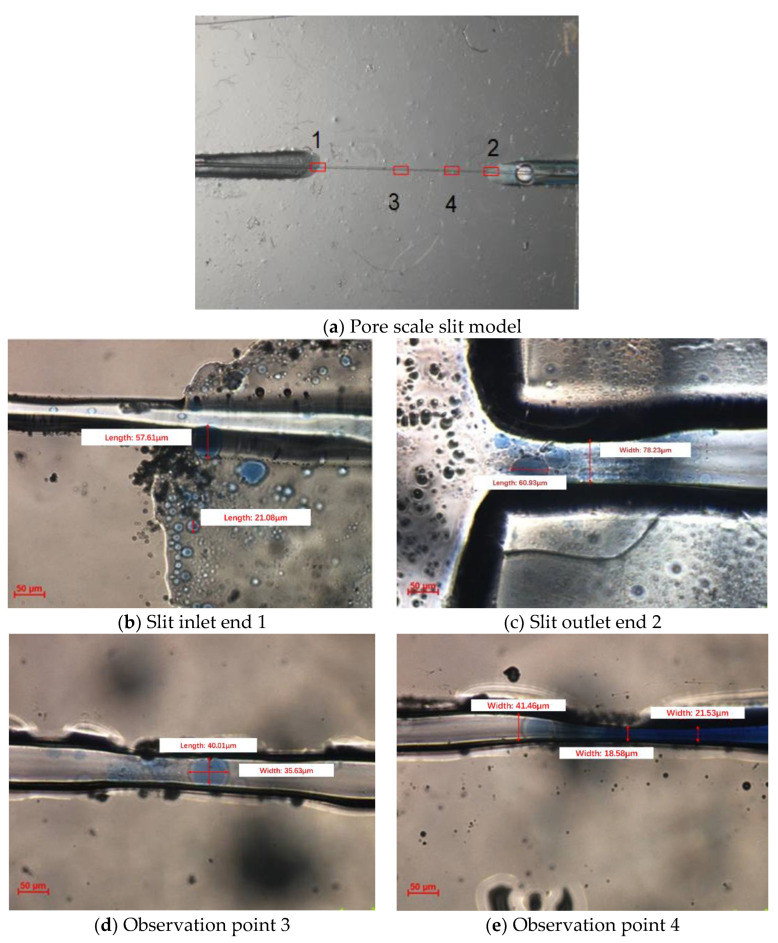
Photos of the microscopic experiment of pore scale slit model.

Figure 3 shows the mechanism of shear thinning of non-Newtonian fluid, including matchstick particles, chain molecules, flexible particle dispersion such as SMG aqueous dispersion system, and molecular line group. Theoretically, flexible particle dispersion is characterized by shear thinning. The reason is that the flexible particle dispersion is composed of multiple components. At rest, all these substances will maintain an internal irregularity and thus have a fairly high internal resistance, that is, a high viscosity, which in turn hinders movement, as shown in Figure 3a. As the shear rate increases, spherical particles or molecular lines suspended in the liquid can more easily slip past each other, turning the spherical particles into rugby bodies, or ellipsoids with smaller diameters, allowing the samples to flow faster, as shown in Figure 3b. SMG aqueous dispersion of low concentration has low viscosity, and the value is close to water, as shown in Figure 4.

### 2.3. Micro-Scale Experiment of SMG

Figure 4 shows the micro-scale physical simulation experiment photos of the pore scale slit model. The pore scale slit model contains four observation points, namely, the inlet end 1, the outlet end 2, and the observation points 3 and 4, as shown in Figure 4a. Observation point 3 is a capillary channel, and observation point 4 is an expanding-shrinking-expanding channel. Because SMG particles flow too quickly through the pores in the injecting process of SMG aqueous dispersion, there is no way to capture images of rapid SMG particle migration directly using a biological microscope. With the injection pump turning off, the velocity of SMG particles decreases. The remaining SMG particles at the inlet end continue to migrate in the microscopic model under the action of inertial forces, which can be recorded by a biological microscope. The flow process of SMG aqueous dispersion is observed with a biological microscope, as shown in Figure 4b–e. Through observation of points 3 and 4, the deformation of SMG particles and the process of temporary plug throat can be clearly observed. In addition, SMG particles observed at outlet 4 show no crushing phenomenon, indicating that SMG particles had good elastic deformation and shear resistance.

**Figure 5:**
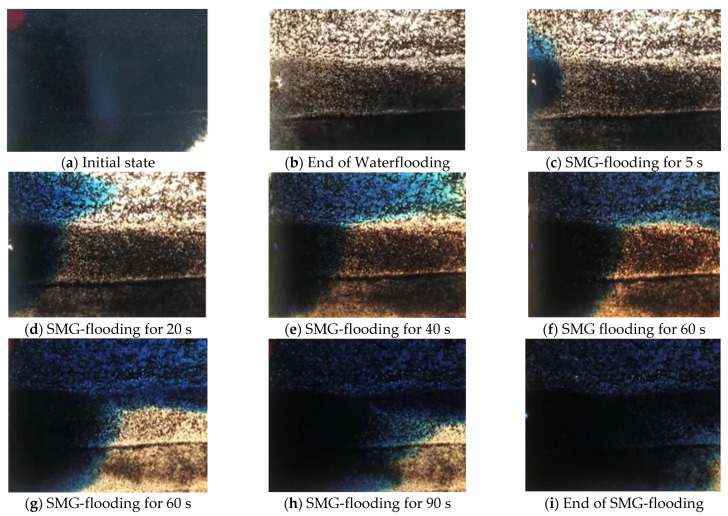
The SMG profile control and oil displacement experiment results for the visual sand inclusion model.

Figure 5 shows the SMG profile control and oil displacement experiment process of the anti-rhythm sand inclusion model. The model is composed of three layers, from top to bottom, the high permeability layer, the middle permeability layer, and the low permeability layer. Figure 5a shows the model after saturated oil. Figure 5b shows the distribution of residual oil when the water-cut at the outlet reaches 95%. In the process of water flooding, the majority of injected water flows into the high permeability layer above, and most of the oil in the high permeability layer and middle permeability layer is displaced. In contrast, the fraction of water flows into a low permeable zone and the lower part of the medium permeability zone, and only a small amount of oil is displaced. Figure 5c shows the initial state of the model at the beginning of SMG flooding. It can be seen from this photo that the seepage direction of SMG is along the main flow line, and part of it flows into the high permeability reservoir. In addition, as can be seen from Figure 5d, most SMG flows into the upper part of the high permeability layer, some enter into the middle permeability layer, and only a small amount enters into the low permeability layer.

Figure 5e,f shows the state of SMG aqueous dispersion just breaking through the high permeability layer. After breaking through the high permeability layer, the SMG aqueous dispersion migrates along the middle and low permeability layers. Figure 5g–i shows the experimental process of SMG improving the contradictions in the layer and starting the middle and low permeability permeable layer.

It can be seen from the residual oil saturation in the model that the oil displaced by SMG flooding is mainly from the middle and low permeability layers. This is because the SMG particle has a certain blocking ability, which makes the water turn to the middle and low permeability layer, which can improve the recovery of the middle and low permeability layer.

### 2.4. Analysis of Experimental Results of the Matching Relationship between SMG Particles and Reservoirs

**Figure 6:**
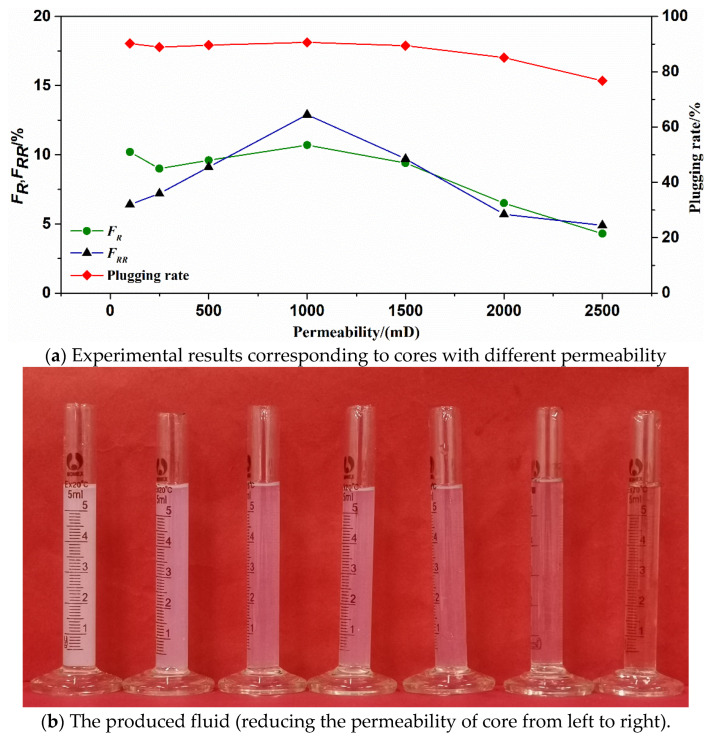
The experiment results of matching between SMG-μm and reservoir.

**Figure 7:**
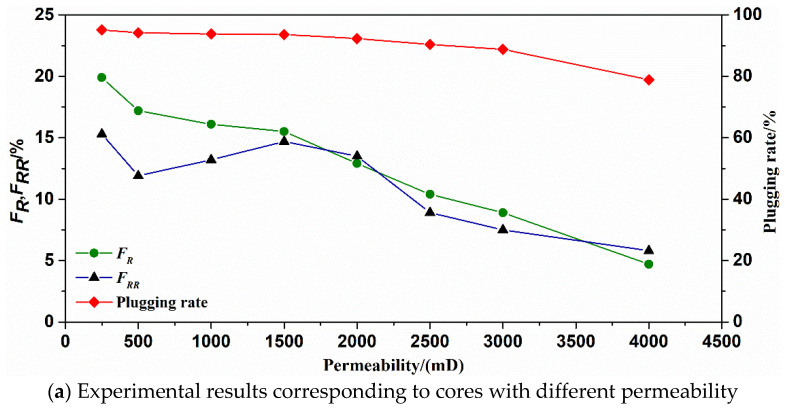

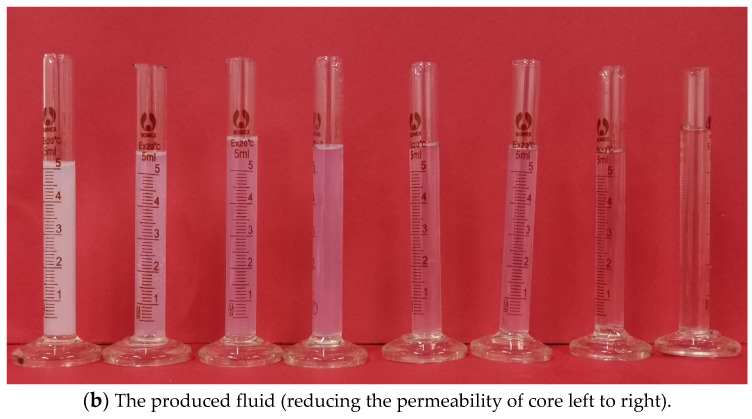
The experiment results of matching between SMG-mm^−^ and reservoir.

The matching relationship, SMG particles and reservoirs can be determined by the displacement experiments of cores. The results are shown in Table 2 and Table 3, respectively. It can be observed from Figure 6a and Figure 7a that the matching coefficient of SMG-μm and SMG-mm^−^ decreases with the increase of core permeability. When the core permeability increases from 100 to 2500 mD, the matching coefficient of SMG-μm decreases from 1.69 to 0.55. When the core permeability increases from 250 to 3000 mD, the corresponding matching coefficient of SMG-mm^−^ decreases from 2.67 to 0.88. As shown in Figure 6b and Figure 7b, with the continuous increase of the matching coefficient, the color of the produced liquid during the injection of the SMG-μm and SMG-mm^−^ gradually becomes lighter, which preliminarily indicates that the SMG concentration in the produced liquid increased with the increasing of core permeability. At the same time, the corresponding resistance factor (*F_R_*) in the displacement experiments of the core decreased with the increase of core permeability. The residual resistance factor (*F_RR_*) first increases and then decreases as the core permeability increases, and the plugging rate increases with the core permeability.

**Figure 8:**
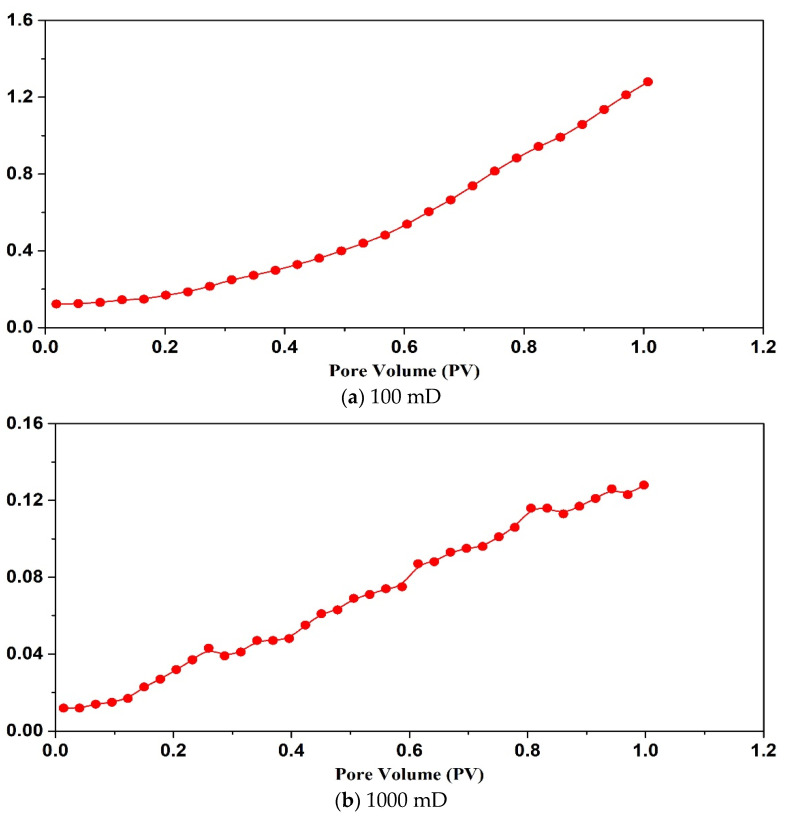

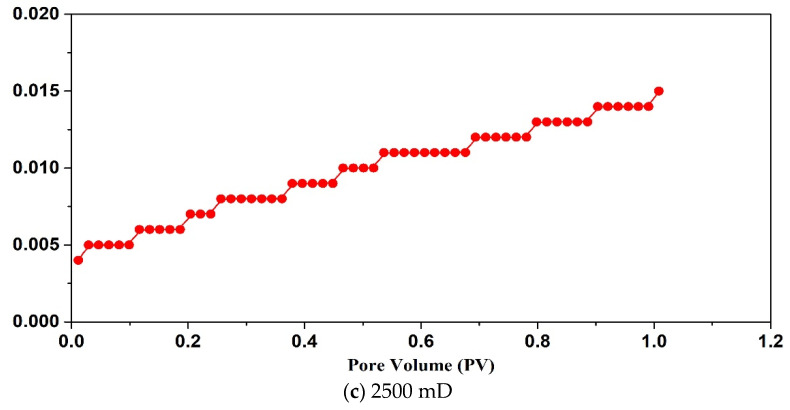
The pressure curve the SMG-μm aqueous dispersion injection stage.

**Figure 9:**
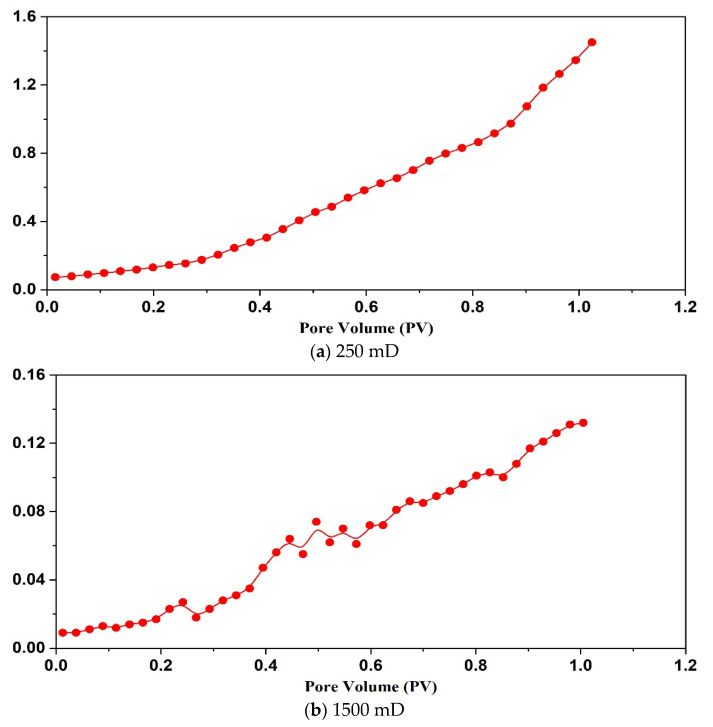

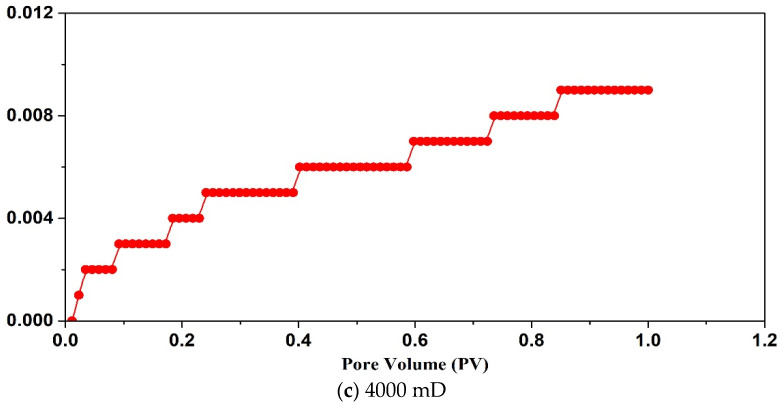
The pressure curve of the SMG-mm^−^ aqueous dispersion injection stage.

By analyzing Figure 8 and Figure 9, it can be seen that when the permeability of the core is less than 100 mD, the SMG-μm particle can’t effectively enter the core and accumulates at the injection end of the core, showing that the injection pressure is an exponential function. When the core permeability is 1000 mD, SMG-μm particle effectively enters the core and migrates into the core, showing a wave-like rise in pressure. When the permeability is 2500 mD, and only if there is enough SMG-μm particle in the throat, the pore throat can be effectively sealed, which is manifested as a slow rise in pressure in a stepwise manner. Similarly, the variation tendency of the injection pressure curve of SMG-mm^−^ particles in the three cores with different permeability of high, medium, and low is basically the same as that of the SMG-μm particle.

Based on the above experimental analysis, the matching results of the relationship between SMG particles and reservoirs can determine that the SMG-μm is best adapted to reservoirs with a permeability of 250–2000 mD, and the corresponding matching coefficient is 0.65–1.40. The SMG-mm^−^ is best adapted to reservoirs with a permeability of 500–2500 mD, and the corresponding matching coefficient is 1.17–2.07.

## 3. Conclusions

SMG has low viscosity and high shear resistance, which makes it easy to migrate deeper into the reservoirs and provides better water-flooding swept control performance.

The microscopic oil displacement mechanism for SMG has been further proved by the visual sand inclusion model. The behavior of “temporary plugging-migration-temporary re-plugging” in the pores is observed, which can expand the water-flooding sweep volume and improve the reservoir’s ultimate oil recovery.

The exact matching relationship between the SMG particle and the reservoir has been determined. SMG-μm is more suitable for reservoirs with 250–2000 mD, and SMG-mm is more suitable for reservoirs with 500–2500 mD.

## 4. Materials and Methods

### 4.1. Preparation of Materials

**Figure 10:**
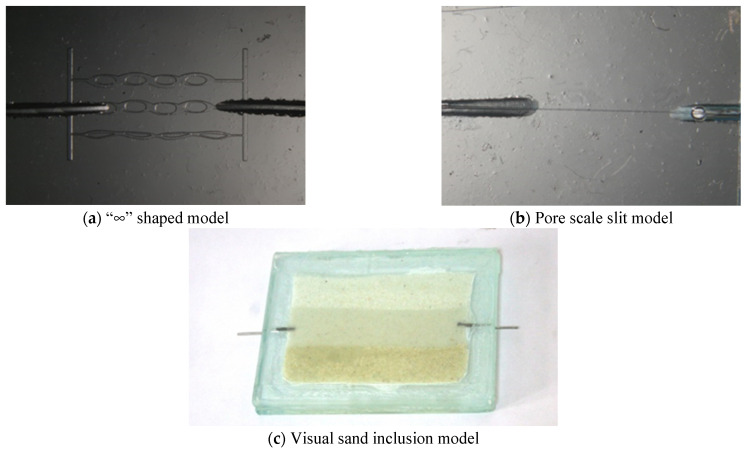
Three test models. (**a**) “∞” shaped model, (**b**) Pore scale slit model, (**c**) Visual sand inclusion model.

Two glass models are developed: pore scale slit model and visual sand inclusion model. (1) The “∞” shape model produced by the traditional microscopic model making process has a throat of 100–200 μm and a depth of about 20 μm, which has the problem of “too large width to depth ratio”, as shown in Figure 10a. In order to prevent SMG from getting stuck in the model, the pore scale slit model is designed and made independently by using glass material. The depth and width of the slit are 90 μm and 10–50 μm, respectively, as shown in Figure 10b. (2) The visual inclusion sand model is formed by filling quartz sand glue between two flat glasses. The model is divided into three layers according to the formation prosodic. From top to bottom, the first layer is filled with 40–60 mesh sand (high permeability layer), the second layer with 80–120 mesh sand (medium permeability layer), and the third layer with 160–180 mesh sand (low permeability layer). The thickness of the sand layer is about 1.3–1.4 mm. The permeability of the three layers is 3000, 1500, and 900 mD, respectively, and the experimental model size is about 13 × 9 × 0.1 cm^3^, as shown in Figure 10c.

The parameters of the artificial core used in experiments are shown in Table 4.

The reagent of micro-SMG (SMG-μm) and submillimeter SMG (SMG-mm^−^) is provided by the Research Institute of Petroleum Exploration and Development of CNPC. In order to exclude the influence of impurity and observe the SMG particle morphology better, the SMG solution is added into a beaker of alcohol with a concentration of 99.7% while stirring with a glass rod to force SMG into full contact with the alcohol. Then, the separable solid of SMG is extracted by repeatedly filtering and washing. Then the extracted separable solid of SMG is placed in an oven at 60 °C for 5 h to obtain SMG dry powder. SMG aqueous dispersion is prepared with SMG dry power and simulated brine. The viscosity of simulated oil is 12 mPa·s. Simulated brine is prepared according to Table 5.

### 4.2. Experimental Setups

**Figure 11:**
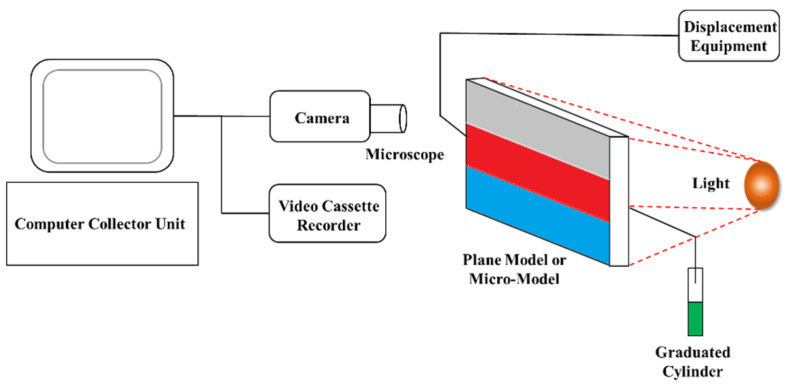
Schematic diagram of plane model and micro-model visual displacement equipment.

The HAAK-RS-600 rheometer with double-gap rotary cylinder sensing system comes from HAAKE Company, Karlsruhe, Germany. The visual displacement equipment of the model includes a stereo microscope, Biooptical Microscope (Nikon 80i), camera, video cassette recorder (VCR), displacement equipment, core holder, intermediate container, pressure sensor, and so on, as shown in Figure 11.

### 4.3. Methods

#### 4.3.1. Measurement of SMG Particle Size and Rheological Property

The dynamic light scattering method (DLS) is used to measure the size of SMG particles. The SMG is stained with methylene blue, and the morphology of SMG is observed by microscope. The concentration of SMG aqueous dispersion is 3000 mg/L. The experimental temperature is 25 °C

The surface viscosity of SMG aqueous dispersion is measured when the shear rate increased from 1 s^−1^ to 80 s^−1^. The test temperature and SMG aqueous dispersion concentration are 25 °C and 3000 mg/L, respectively.

#### 4.3.2. Microinjection Experiment

The visual sand inclusion model is weighed (W_1_) after placing it in a 60 °C oven for 12 h. Subsequently, this model is weighed (W_2_) again after being evacuated and saturated simulated brine. The saturated brine volume can be calculated by V = W_2_ − W_1_, which can reflect the pore volume of the sand inclusion model. Besides, the original oil saturation of the model is established by injecting simulated oil into this model. Water-flooding of the visual sand inclusion model is performed after 48 h of oil saturation. The displacement velocity is 0.3 mL/min, and the changes of oil and water production at different times are recorded. When the water-cut of the produced liquid reaches about 95%, water-flooding is stopped, and SMG aqueous dispersion is injected. The injection volume is 0.3 times the pore volume (PV) of the model. It is emphasized that the concentration of SMG aqueous dispersion is 3000 mg/L. The experimental temperature is 25 °C.

#### 4.3.3. Experiment on the Matching Relationship between SMG Particle and Reservoir

The artificial cores are successively dried, vacuumed, saturated with simulated brine, and permeability measured. Subsequently, the matching experiments between SMG particles and the reservoir is conducted. The concentration of SMG aqueous dispersion is 3000 mg/L. The specific operation steps are as follows: (1) Primary water-flooding, brine is injected into the artificial core until the pressure is stable; (2) SMG-flooding, 1 time the pore volume of the SMG aqueous solution is injected into the core; (3) Subsequent water-flooding, the experiment is terminated when the injection pressure shows no change for 20 min. It is emphasized that the injection rate of the whole experiment is 1 mL/min. The pressures at end of primary water flooding, SMG-flooding, and subsequent water flooding are *P*_1_, *P*_2_, and *P*_3_, respectively. The resistance factor (*F_R_*) and residual resistance factor (*F_RR_*) can be calculated with Equations (4) and (5). The confining pressure of the core holder is always greater than the injection pressure by about 3 MPa. The concentration of SMG aqueous dispersion is 3000 mg/L. The experimental temperature is 25 °C.

(4) $$F_{R}=\frac{P_{2}}{P_{1}}$$

(5) $$F_{RR}=\frac{P_{3}}{P_{1}}$$

The ratio of the SMG median particle diameter to the average pore throat diameter of the reservoir or core is defined as the matching coefficient *Z*, as shown in Equation (6), where the average pore throat diameter of the reservoir is based on the Cozeny-Kalman formula [34], as shown in Equation (7). By combining Equations (6) and (7), Equation (8) can be obtained.

(6) $$Z=\frac{d_{SMG}}{d_{throat}}$$

(7) $$K=\frac{\varphi {d_{throat}}^{2}}{16f_{ck}\tau^{2}}$$

(8) $$Z=\sqrt{\varphi {d_{SMG}}^{2}/(72K)}$$

where $d_{SMG}$ is SMG median particle diameter, μm; $d_{throat}$ is the average pore throat diameter of the reservoir or core, μm;φ is porosity of reservoir or core, %. K is permeability. $f_{ck}\tau^{2}$ is the constant, which is related to the complexity of the pore throat structure. In this paper, the value of $f_{ck}\tau^{2}$ is 4.5.

## Figures and Tables

**Table 1:** Swelling property of SMG.

Type	*d*_0_, μm	*d_max_*, μm	B = (*d_max_* − *d*_0_)/*d*_0_
SMG-μm	3.2	28.6	7.9
SMG-mm^−^	16.6	68.3	3.1

**Table 2:** The matching results between SMG-μm and reservoir.

Permeability/(mD)	Matching Coefficient	*F_R_*	*F_RR_*	Plugging Rate
100	1.69	10.2	6.4	90.2
250	1.40	9.0	7.2	88.9
500	1.23	9.6	9.1	89.6
1000	0.98	10.7	12.9	90.6
1500	0.83	9.4	9.7	89.4
2000	0.65	6.5	5.7	85.1
2500	0.55	4.3	4.9	76.7

**Table 3:** The matching results between SMG-mm^−^ and reservoir.

Permeability/(mD)	Matching Coefficient	*F_R_*	*F_RR_*	Plugging Rate
250	2.67	19.9	15.3	95.1
500	2.07	17.2	11.9	94.2
1000	1.67	16.1	13.2	93.8
1500	1.55	15.5	14.7	93.6
2000	1.32	12.9	13.5	92.3
2500	1.17	10.4	8.9	90.4
3000	0.88	8.9	7.5	88.8
4000	0.75	4.7	5.8	78.9

**Table 4:** The parameter for the artificial core.

Core Number	Length/cm	Diameter/cm	Porosity/%	Permeability/mD
2-1	10.05	2.51	18.5	100
2-2	10.03	2.50	20.1	250
2-3	10.05	2.52	22.0	500
2-4	10.01	2.51	24.8	1000
2-5	10.02	2.50	25.8	1500
2-6	10.03	2.51	26.6	2000
2-7	10.02	2.50	29.1	2500
3-1	10.04	2.53	22.2	250
3-2	10.02	2.53	23.8	500
3-3	10.03	2.50	24.8	1000
3-4	10.02	2.51	26.8	1500
3-5	10.05	2.52	27.4	2000
3-6	10.06	2.53	27.9	2500
3-7	10.08	2.51	29.5	3000
3-8	10.10	2.51	32.9	4000

**Table 5:** The ionic composition of the stimulated brine.

Ionic Composition	Na^+^, K^+^	Ca^2+^	Mg^2+^	CO_3_^2−^	HCO_3_^−^	SO_4_^2−^	Cl^−^	TDS
Concentration, mg/L	3091.96	276.17	158.68	14.21	311.48	85.29	5436.34	9374.13

## Data Availability

The data supporting the findings of this manuscript are available from the corresponding authors upon reasonable request.
